# Aging and the aggregating proteome

**DOI:** 10.3389/fgene.2012.00247

**Published:** 2012-11-20

**Authors:** Della C. David

**Affiliations:** German Center for Neurodegenerative Diseases (DZNE)Tübingen, Germany

**Keywords:** protein aggregation, aging, protein homeostasis, *C. elegans*, neurodegeneration, chaperones

## Abstract

For all organisms promoting protein homeostasis is a high priority in order to optimize cellular functions and resources. However, there is accumulating evidence that aging leads to a collapse in protein homeostasis and widespread non-disease protein aggregation. This review examines these findings and discusses the potential causes and consequences of this physiological aggregation with age in particular in relation to disease protein aggregation and toxicity. Importantly, recent evidence points to unexpected differences in protein-quality-control and susceptibility to protein aggregation between neurons and other cell types. In addition, new insight into the cell-non-autonomous coordination of protein homeostasis by neurons will be presented.

## NATURE OF PROTEIN AGGREGATION IN DISEASE

Protein aggregation is the common defining feature in neurodegenerative diseases such as Alzheimer’s and Parkinson’s disease as well as systemic amyloidosis. In these diseases, one or several distinct aggregation-prone polypeptides become misfolded and are packed into large insoluble hallmark structures. Disease aggregation affects proteins with very different native structures. For example, natively unfolded proteins such as tau and β-amyloid aggregate in Alzheimer’s disease whereas globular proteins rich in β-sheets like transthyretin, rich in α-helices such as apolipoprotein A1 or containing both β-sheets and α-helices such as gelsolin aggregate in different types of systemic amyloidosis (Uversky et al., 2006). Despite these differences, X-ray diffraction results suggest that all these proteins adopt a very specific amyloid structure in the aggregates where they are stacked together in cross-β-sheets parallel to the fibril axis (Eisenberg and Jucker, 2012). Aggregates typically contain amyloid fibrils which grow at their ends by providing a template for the addition of further monomers. Soluble aggregation intermediates have also been identified, in particular prefibril and fibril oligomers which are recognized by different antibodies (Glabe, 2008). These structures are more reactive than the long fibrils and are generally considered more toxic to the organism. Although aggregates often contain different proteins, amyloid fibrils and oligomers are classically composed of identical proteins.

## THE PROTEOME ON THE EDGE OF SOLUBILITY

The causes, consequences, and regulation of disease protein aggregation have been extensively discussed in other reviews (Soto, 2003; Ross and Poirier, 2005; Douglas and Dillin, 2010; Eisenberg and Jucker, 2012). The present mini-review will focus on recent evidence related to the disruption of protein homeostasis with age leading to widespread protein insolubility and aggregation in the absence of disease. Indeed, it is predicted that all proteins have the capacity to aggregate under specific conditions. For example, changes in pH, heating, denaturing conditions, or increased protein concentrations all tend to favor aggregation. Recently, Goldschmidt et al. (2010) predicted that the majority of proteins have short self-complementary sequences, which can initiate the formation of a steric zipper structure thus promoting aggregation. Normally, aggregation is avoided by burying these aggregation-prone regions inside the protein during the folding process. However, partial unfolding could be sufficient to uncover these regions and lead to aggregation (Chiti and Dobson, 2009).

Computational analysis indicates that the proteome is only marginally stable (Ghosh and Dill, 2010). Cells have likely optimized protein expression levels to prevent aggregation, leaving thereby little space for deviations in concentration (Tartaglia et al., 2007; Tartaglia and Vendruscolo, 2009). Indeed, this delicate balance can be easily disrupted. For example, exposing cells in culture to thermal stress prompts protein insolubility (Salomons et al., 2009). Artificially inducing macromolecular crowding coupled with increased ionic strength after exposure to high salt concentrations leads to widespread protein insolubility and rapid irreversible protein aggregation in the model organism *Caenorhabditis elegans* (Burkewitz et al., 2011).

## DECREASED PROTEIN-QUALITY-CONTROL WITH AGE

In a healthy young organism, several layers of quality-control help proteins to remain functional and prevent aggregation (Balch et al., 2008). This starts with the regulation of transcriptional and translational rates as well as a tight control over the folding of newly synthesized proteins by providing different chaperones to assist the folding process (Hartl et al., 2011). After a damaged protein is deemed beyond repair, it is targeted by chaperones to the proteasomal or autophagy degradation systems (Kettern et al., 2010). In addition to the cytoplasmic protein-quality-control components, organelle-specific quality-control systems have been identified in the nucleus, endoplasmic reticulum, and mitochondria (Sidrauski et al., 1998; Haynes and Ron, 2010; Rosenbaum and Gardner, 2011). As the organism ages, this regulation of protein homeostasis becomes disrupted. In *C. elegans*, a sharp decrease in chaperone expression is correlated with the end of the reproductive phase and leads to the aggregation of folding-defective mutant proteins (Ben-Zvi et al., 2009). In mammals, the unfolded protein response activated by ER stress is impaired with age (Brown and Naidoo, 2012). Furthermore, aging is associated with a decline in proteasome activity in a variety of tissues in rats (Anselmi et al., 1998; Keller et al., 2000). Similarly, lysosomal chaperone-mediated autophagy activity is reduced in old-aged rat livers and senescent human fibroblasts (Cuervo and Dice, 2000). Conversely, enhancing lysosomal degradation as well as overexpressing RPN11, one of the 19S proteasome subunits, suppresses disease-related protein aggregation (Tonoki et al., 2009; Yang et al., 2011). Furthermore, aging is also associated with increased oxidative stress, leading to irreversible oxidation and nitration of proteins, which impairs their degradation (Squier, 2001; Poon et al., 2006). Errors during transcription and translation could provide a further challenge to the protein-quality-control system with age (Gidalevitz et al., 2010). In addition, molecular misreading during transcription causing dinucleotide deletions plays a role in Alzheimer’s and Huntington’s disease (van Leeuwen et al., 1998; Lam et al., 2000; de Pril et al., 2004). All these changes with age could contribute to widespread protein aggregation.

## IDENTIFYING THE AGE-RELATED AGGREGATING PROTEOME

Although protein homeostasis is disrupted with age, it was unclear to what extent this affects the stability of the proteome (Morimoto and Cuervo, 2009). Recently, increased levels of protein hydrophobicity were detected in brains from aging rats which could promote protein aggregation (Chiti and Dobson, 2006; Dasuri et al., 2010). Consequently, a study with *Drosophila* revealed the accumulation of aggregated proteins with age in different tissues (Demontis and Perrimon, 2010). These aggregated structures were detergent insoluble and appeared to be filamentous by electron microscopy, two features associated with disease aggregation. Independently, two groups set out to identify the age-related aggregating proteome in *C. elegans* using mass-spectrometry (David et al., 2010; Reis-Rodrigues et al., 2012). *C. elegans* is widely used to study the aging process as these animals have a relatively short lifespan and show many characteristic aging features observed in higher organisms (Garigan et al., 2002; Kenyon, 2005). To isolate proteins in a similar state to aggregated proteins in disease, both groups adopted sequential biochemical fractionation methods based on differential solubility, which is widely used to extract disease aggregates in the field of neurodegeneration research (Lee et al., 1999). Both groups discovered a substantial increase in the insolubility of several hundred proteins with age confirming a widespread disruption in protein homeostasis. The significant overlap in protein identities and functional categories between both studies shows that aggregation does not randomly affect the whole proteome, but rather a subset of proteins. Furthermore, computational analysis revealed that these aggregation-prone proteins have a higher propensity to form β-sheets, a driving force behind disease protein aggregation. In addition, *in vivo* analysis of several aggregation-prone proteins with fluorescent protein tags consistently showed the abnormal clumping of these proteins into aggregate-like structures where the proteins are in a highly immobile state (David et al., 2010).

Although these physiological age-related aggregates resemble disease aggregates in several aspects, it remains to be determined whether these aggregates are in an amyloid or amorphous state. Interestingly, Alavez et al. (2011) showed that the prefibrillar-oligomeric-specific antibody A11 binds specifically to structures in the aging worm in the absence of disease. This antibody recognizes a conformation characteristic of aggregation intermediates formed by diverse disease-related aggregation-prone proteins such as β-amyloid, α-synuclein, and polyglutamine (Kayed et al., 2003). These intermediates are considered as precursors to larger amyloid fibrils (Lee et al., 2011). Evidence from bacteria also suggests that a variety of proteins can aggregate into an amyloid structure. Indeed, overexpression of exogenous proteins in bacteria often leads to their aggregation and the analysis of these aggregates revealed a partial amyloid structure (Wang et al., 2008). As the authors propose, “there might be no amorphous state of a protein aggregate” and one could speculate that physiological age-related aggregates are composed of a mixture of amyloid and disordered structures.

## THE CONSEQUENCES OF AGE-RELATED PHYSIOLOGICAL AGGREGATION IN NEURODEGENERATIVE DISEASE AND AGING

Aging is the main known risk factor for sporadic neurodegenerative diseases. Henceforth, an important question is whether non-disease protein aggregation may put the brain at risk for aggregation of disease proteins. Proteomic analyses of disease aggregates reveal a large number of proteins that are associated with the main hallmark disease-aggregating protein (Liao et al., 2004; Wang et al., 2005; Xia et al., 2008). Comparison with physiological age-aggregating proteins tells us that a significant proportion of these proteins can aggregate themselves without the presence of disease aggregates. Non-disease protein aggregation could initiate or accelerate disease aggregation by several mechanisms. First, physiological aggregation could titrate anti-aggregation factors away from disease-aggregating proteins. In *C. elegans* body-wall muscles, Gidalevitz et al. (2006) showed that expressing either aggregation-prone polyglutamine or mutated proteins sensitive to misfolding reduces the folding capacity in these cells leading to enhanced protein aggregation. Similarly, widespread protein insolubility caused by heat shock impaired the ubiquitin-dependent proteasomal degradation (Salomons et al., 2009). Second, the aggregation of non-disease-associated proteins could directly induce the aggregation of disease-specific proteins by a cross-seeding mechanism. Exposure of hydrophobic stretches plays an important role in promoting protein aggregation (Munch and Bertolotti, 2010). Recently, Olzscha et al. (2011) found that artificially aggregating proteins preferentially forming oligomers with exposed hydrophobic surfaces caused the most damage to the cell. These artificial aggregating proteins efficiently sequestered cellular proteins into aggregates. Similarly, the misfolding and aggregation of non-disease proteins with age could reveal previously hidden hydrophobic stretches which may promote disease protein aggregation.

It is tempting to speculate on the consequences of physiological protein aggregation in the context of aging. During aging, aggregation affects a large number of proteins, which play a role in regulating protein homeostasis as well as preventing disease protein aggregation (David et al., 2010; Reis-Rodrigues et al., 2012). Sequestration of these proteins into aggregates could lead to a decrease in functional protein available for the cell. In addition, proteins which play a role in determining adult lifespan are over-represented in the pool of aggregation-prone proteins (David et al., 2010). Reis-Rodrigues et al. (2012) showed that reducing the levels of aggregation-prone proteins by RNA interference extends lifespan for nearly half of the candidates tested. Two different possibilities could explain these results. First, protein aggregates or the reactive misfolded proteins during aggregation are toxic for the organism, and by down-regulating expression of the aggregation-prone proteins, the protein homeostasis is restored, which leads to the lifespan extension. Second, the cellular function carried out by aggregation-prone proteins is detrimental during aging and their aggregation may be a protective mechanism. It is impossible to distinguish between these possibilities based on RNA interference experiments alone.

Finally, it remains possible that at least a proportion of physiological aggregation has no negative consequences. Numerous examples of functional aggregation have been discovered (Fowler et al., 2007). To date, 25 proteins have been identified in yeast which can switch to a prion conformation. These proteins tend to be important regulators of gene expression as well as signaling transducers. The targeted loss-of-function caused by their aggregation allows the evolution of new traits in response to environmental changes (Halfmann et al., 2012). Functional aggregation is also found in mammals. For example, peptide hormones are stored in an amyloid aggregate and are released when needed (Maji et al., 2009). Therefore certain physiological aggregation could be a mechanism to store these proteins or rapidly inhibit their function. This type of aggregation could increase with age in response to decreased demand for the active protein or be enhanced by deregulation of the mechanisms responsible for resolubilizing the aggregated proteins.

Overall, it will be important to determine whether physiological protein aggregation contributes to tissue degeneration with age or is merely a consequence of aging. In particular, the dynamics of aggregation may determine whether the net outcome is positive or negative for the organism. Combined with the decline in protein-quality-control with age, aggregation-prone proteins which tend to remain in a misfolded and soluble state would be predicted to be more harmful than those which are rapidly sequestered into compact insoluble aggregates.

## CELLULAR MECHANISMS AVAILABLE TO MANAGE AGE-DEPENDENT PROTEIN AGGREGATES

An intricate protein-quality-control system normally ensures that proteins are properly folded and damaged proteins are quickly removed (Hartl et al., 2011). However, in cases of extreme stress such as proteasome failure or heat stress, a large pool of misfolded proteins rapidly accumulates in the cell and assemblies into aggregates. Throughout evolution, the cell has developed different mechanisms to deal with this aberrant protein aggregation and either resolubilize the proteins or sequester aggregates away from vital functions. In bacteria, inclusions are formed preferentially at the poles upon heat stress and are resolubilized by the AAA+ chaperone ClpB, in collaboration with heat shock protein DnaK (Winkler et al., 2010). In yeast, stress-induced misfolded proteins and amyloidogenic proteins are actively collected in different centers in the cell (Kaganovich et al., 2008). Dependent on the state of misfolding or aggregation propensity, the damaged protein is either ubiquitinated and targeted to a juxtanuclear quality control compartment (JUNQ) or directed into peripheral insoluble protein deposits (IPODs) by Hsp42 (Kaganovich et al., 2008; Specht et al., 2011). Hsp104, the yeast homolog of the prokaryote ClpB, is targeted to stress-induced aggregates by Hsp70 and promotes their disaggregation (Winkler et al., 2012). In animal cells, aggregating proteins induced by stress as well as some disease-aggregating proteins are preferentially sequestered into a structure called the aggresome (Johnston et al., 1998). Here, ubiquitinated aggregates are actively targeted to the aggresome localized at the microtubule-organizing center through the concerted action of dynein and the histone deacetylase HDAC6 (Johnston et al., 2002; Kawaguchi et al., 2003). Of note, non-ubiquitinated aggregates have also been identified in the aggresome (Garcia-Mata et al., 1999; Ben-Gedalya et al., 2011). In addition, JUNQ and IPOD structures have been observed in mammalian cells (Kaganovich et al., 2008; Weisberg et al., 2012). Metazoans lack a direct homolog of Hsp104. However, recent studies demonstrate that Hsp110 in concert with Hsp70–Hsp40 and small Hsps actively resolubilize both heat-induced and disease aggregates in metazoans (Duennwald et al., 2012; Rampelt et al., 2012).

It remains to be shown whether any of these mechanisms are involved in managing age-dependent protein aggregation. Interestingly, physiological protein aggregation has been identified in different locations in the cell including the nucleus and does not necessarily co-localize with disease-protein aggregates (David et al., 2010).

## DIFFERENCES BETWEEN NEURONAL AND NON-NEURONAL REGULATION OF PROTEIN HOMEOSTASIS

In the context of disease, different tissues and cell-types are susceptible to protein aggregation. For example in patients with sporadic inclusion-body myositis, amyloid-β and tau protein aggregate exclusively in muscles together with several other proteins (Askanas et al., 2009). Conversely, in Alzheimer’s disease, amyloid-β and tau aggregates are restricted to brain tissue. The reasons for this specific vulnerability remain unclear. Physiological age-related aggregates have been identified in all tissues examined including neurons. Tissue susceptibility to age-related aggregation will probably be further refined by examining more individual aggregation-prone proteins. Interestingly, results from *C. elegans* and *Drosophila* would suggest that neurons are to some extent more resistant to age-dependent protein insolubility and aggregation than muscles (David et al., 2010; Demontis and Perrimon, 2010). How could this be? Recent results show that neurons and muscles have developed different strategies to deal with protein misfolding, which change with age. Using luciferase aggregation and the subsequent recovery of luciferase activity after heat shock, Kern et al. (2010) searched for differences in chaperone capacity in neurons and muscle cells of young and aged *C. elegans*. They found that young muscles efficiently prevented protein aggregation but lost this activity with age. On the other hand, young neurons have a delayed chaperone response but compensate by increasing disaggregation and refolding activity. With age, neurons switched to the strategy used by young muscle cells in that they actively prevent aggregation but no longer promote refolding. In contrast, old muscle cells become highly susceptible to protein misfolding and aggregation. Similarly, Hamer et al. (2010) observed that the proteasomal degradation capacity differs between muscles and neurons. Using a photoconvertible fluorescent reporter marked for degradation by ubiquitin, the authors show that young neurons rapidly removed ubiquitinated proteins through the proteasome, whereas muscles only slowly degraded proteins. Higher protein turn-over in young neurons is achieved by improving substrate recognition using the ubiquitin-binding proteasome subunit RPN10. Interestingly, the degradation rate varies greatly between neuronal cell types which may help explain differences in neuronal susceptibility to protein homeostasis disruption. With age, the rate of protein degradation decreased solely in neurons while still remaining higher than in muscles. Overall, these different strategies used by neuronal and non-neuronal cells to control protein homeostasis and how they are modified to compensate during aging may render them more or less susceptible to physiological protein aggregation.

## NON-AUTONOMOUS CONTROL OF PROTEIN AGGREGATION

Neurons play an important role in coordinating protein homeostasis regulation throughout the organism in response to changes in the environment. How does this affect physiological protein aggregation in different tissues? In *C. elegans*, thermosensory AFD neurons initiate activation of the transcription factor HSF-1, driving the transcription of chaperones, in non-neuronal tissues in response to acute heat stress (Prahlad et al., 2008). However, in the absence of heat stress, these same neurons prevent the up-regulation of chaperones in non-neuronal tissues in response to chronic protein damage and aggregation (Prahlad and Morimoto, 2011). Therefore under normal conditions, *C. elegans* blunts its protein folding machinery and cannot appropriately respond to protein aggregation. Neurons potentially also play a role in coordinating the mitochondrial unfolded protein response in non-neuronal tissues. Indeed, Durieux et al. (2011) found that mitochondrial impairment only in neurons induces the mitochondrial unfolded protein response in the intestine. Furthermore, excessive neuronal signaling through cholinergic motor neurons leads to increased misfolding of folding-defective proteins and aggregation of polyglutamine in muscle cells (Garcia et al., 2007). Therefore, depending on the circumstances, neurons can modulate protein homeostasis in both directions, either by promoting or inhibiting protein aggregation.

On the other hand, the state of protein homeostasis in non-neuronal tissue can influence neuronal protein health. Indeed, up-regulating Pten/FOXO signaling specifically in fly muscles reduces the release of insulin-like peptides from the brain, which prevented age-dependent protein aggregation in the brain and other tissues (Demontis and Perrimon, 2010).

## OUTLOOK

The extensive identification of proteins aggregating during aging provides us with a starting point to understand the collapse in protein homeostasis with age. It will be essential to integrate our vast knowledge on protein homeostasis regulation to identify the key factors controlling physiological protein aggregation during the aging process. Delaying aging by dietary restriction or reducing insulin/IGF-1 signaling has been shown to mitigate the proteotoxicity of disease-protein aggregation in invertebrates and mammals (Morley et al., 2002; Cohen et al., 2006, 2009; Steinkraus et al., 2008; Freude et al., 2009; Killick et al., 2009; Teixeira-Castro et al., 2011; Zhang et al., 2011). Similarly, reducing insulin/IGF-1-like signaling (David et al., 2010; Demontis and Perrimon, 2010) or using chemical compounds such as thioflavin (Alavez et al., 2011) tell us that it is also possible to modulate physiological age-related protein aggregation (also see review Alavez and Lithgow, 2011). In both *C. elegans* and *Drosophila*, age-dependent protein aggregation occurs without additional stresses or overexpression of exogenous proteins. Compared to expressing human disease-aggregating proteins in these models, examining age-dependent aggregation gives us an unparalleled opportunity to discover new physiological pathways that control aggregation. Particularly, it will be important to investigate the interplay between physiological and disease protein aggregation. A major goal will be to translate these findings into a mammalian system and use this knowledge to develop therapies to promote healthy aging in humans.

## Conflict of Interest Statement

The author declares that the research was conducted in the absence of any commercial or financial relationships that could be construed as a potential conflict of interest.
